# The contribution to policies of an exposome-based approach to childhood obesity

**DOI:** 10.1093/exposome/osad006

**Published:** 2023-05-26

**Authors:** Paolo Vineis, Evangelos Handakas, Rossella Alfano, Christopher Millett, Daniela Fecht, Leda Chatzi, Michelle Plusquin, Tim Nawrot, Lorenzo Richiardi, Henrique Barros, Martine Vrijheid, Franco Sassi, Oliver Robinson

**Affiliations:** 1 MRC Centre for Environment and Health, School of Public Health, Imperial College, London, UK; 2 Centre for Environmental Sciences, Hasselt University, Diepenbeek, Belgium; 3 Public Health Policy Evaluation Unit, School of Public Heath, Imperial College London, London, UK; 4 NIHR Health Protection Research Unit in Chemical Radiation Threats and Hazards, School of Public Health, Imperial College London, London, UK; 5 Department of Population and Public Health Sciences, University of Southern California, Los Angeles, CA, USA; 6 Cancer Epidemiology Unit, Department of Medical Sciences, University of Turin, Turin, Italy; 7 Departamento de Ciências da Saúde Pública e Forenses e Educação Médica, Faculdade de Medicina, Universidade do Porto, Porto, Portugal; 8 Institute for Global Health (ISGlobal), Barcelona, Spain; 9 Department of Medicine and Life Sciences, Universitat Pompeu Fabra (UPF), Barcelona, Spain; 10 Consorcio de Investigación Biomédica en Red de Epidemiología y Salud Pública (CIBERESP), Madrid, Spain; 11 Centre for Health Economics and Policy Innovation, Department of Economics and Public Policy, Imperial College Business School, London, UK; 12 Mohn Centre for Children’s Health and Well-being, School of Public Health, Imperial College London, London, UK; 13 NOVA National School of Public Health, Public Health Research Center, Comprehensive Health Research Center, CHRC,, NOVA University Lisbon, Lisbon, Portugal

**Keywords:** metabolomics, meet-in-the-middle, branched chain amino acids, policy, multi-omics, adiposity

## Abstract

Childhood obesity is an increasingly severe public health problem, with a prospective impact on health. We propose an exposome approach to identify actionable risk factors for this condition. Our assumption is that relationships between external exposures and outcomes such as rapid growth, overweight, or obesity in children can be better understood through a “meet-in-the-middle” model. This is based on a combination of external and internal exposome-based approaches, that is, the study of multiple exposures (in our case, dietary patterns) and molecular pathways (metabolomics and epigenetics). This may strengthen causal reasoning by identifying intermediate markers that are associated with both exposures and outcomes. Our biomarker-based studies in the STOP consortium suggest (in several ways, including mediation analysis) that branched-chain amino acids (BCAAs) could be mediators of the effect of dietary risk factors on childhood overweight/obesity. This is consistent with intervention and animal studies showing that higher intake of BCAAs has a positive impact on body composition, glycemia, and satiety. Concerning food, of particular concern is the trend of increasing intake of ultra-processed food (UPF), including among children. Several mechanisms have been proposed to explain the impact of UPF on obesity and overweight, including nutrient intake (particularly proteins), changes in appetite, or the role of additives. Research from the Avon Longitudinal Study of Parents and Children cohort has shown a relationship between UPF intake and trajectories in childhood adiposity, while UPF was related to lower blood levels of BCAAs. We suggest that an exposome-based approach can help strengthening causal reasoning and support policies. Intake of UPF in children should be restricted to prevent obesity.

## Introduction

Childhood overweight and obesity are increasing in most of the world, and this trend hampers the health of future generations, since obesity in childhood leads to poor aging and an increased risk of chronic diseases in adulthood. Programmes to prevent childhood obesity have been so far mainly school-based, and effects have been limited, with the best results obtained in younger children. Such programs have almost entirely focused on behavior-oriented prevention. Structural interventions that support behavior change at the population level include, for example, taxes on unhealthy foods and standards for meals. Also, breastfeeding is recommended.^1^

However, the causes of childhood obesity are still largely unknown, though it is likely that maternal and own diet play a key role. Diet is a very complex exposure, and disentangling the effects of different components is not straightforward. Here, we propose an exposome approach to identifying actionable risk factors for childhood obesity. The justification for the exposome approach includes three steps: an examination of different dietary patterns and other risk factors for childhood obesity and overweight (external exposome); the investigation of internal molecular changes through agnostic metabolomic and epigenetic studies (internal exposome); and the connection between the two, that is, the identification of molecular pathways that link the external exposome and the outcomes via the internal exposome. The latter step is expected to contribute to a causal interpretation of statistical associations between exposures and outcomes and thus support policies.

This article draws on literature reviews and evidence mainly (but not exclusively) from the STOP consortium funded by the European Commission (https://www.stopchildobesity.eu/). The consortium included six different birth cohorts from different countries: INMA (Spain), Rhea (Greece), Piccolipiù (Italy), Generation XXI (Portugal), ENVIRONAGE (Belgium), Avon Longitudinal Study of Parents and Children (ALSPAC) (United Kingdom), and HELIX (EU). These studies collected data on parental and children’s behavior, anthropometric data, and blood and urine samples that were analyzed with metabolomics, epigenetics, and relevant biomarkers. The aim of this article is to use an exposome approach to identify diet-related pathways that are supported by reasonably sound evidence, are biologically plausible, and are actionable, in order to guide prevention of obesity in children.

## The evidence so far

### Risk factors

We focus on risk factors for childhood obesity at two general time-periods: early life, with a focus on prenatal exposures, and those risk factors during childhood and adolescence that constitute the “obesogenic environment”.

For early-life risk factors, we build upon background knowledge coming from previous epidemiological studies on the main risk factors for children obesity in the first 1000 days of life. A systematic review conducted by others examined 282 studies that met the inclusion criteria.^2^ They found risk factors during the first 1000 days that were consistently associated with later childhood obesity, including *higher maternal pre-pregnancy BMI*, *prenatal tobacco exposure, maternal excess gestational weight gain, high infant birth weight*, and *accelerated infant weight gain*. A lower degree of evidence was found for gestational diabetes, child care attendance, low strength of maternal–infant relationship, low socioeconomic status, curtailed infant sleep, inappropriate bottle use, introduction of solid food intake before 4 months of age, and infant antibiotic exposure. Uncertainty still surrounds the role of maternal and own dietary risk factors, which is what we aim to clarify in this article.

We are not considering here another important branch of (external) exposome research, the impact of the built environment. The evidence has been reviewed within STOP,^3^ indicating an effect of some characteristics of the built environment on childhood obesity, mainly associated with traffic-related air pollution and characteristics supporting walking. These conclusions were supported by a further STOP study that found that more vegetation, more building density, less population density, and areas without major roads were associated with greater child physical activity.^4^

## Internal exposome: molecular pathways

We considered potential underlying molecular and metabolic pathways, and to this end we conducted two systematic reviews. The first is a systematic review of metabolomic studies of childhood obesity, following the PRISMA guidelines.^5^ A consistent metabolic profile of childhood obesity was observed including *amino acids* (*particularly branched chain—branched-chain amino acid (BCAA)—and aromatic amino acids*), *carnitines, lipids*, and *steroids*. These signatures appear largely concordant with those associated with obesity in adult studies.^6^ We notice that BCAA were cross-sectionally increased in children with obesity, although studies were lacking regarding markers that may predict subsequent development of obesity. There are several limitations in the investigations we have reviewed: few longitudinal studies, limited annotation and metabolite coverage, small sample sizes, and unclear covariate adjustment. While the review highlighted that metabolomic investigations into childhood obesity are a developing field, the metabolic profile of childhood obesity appears to be informative regarding mechanisms underlying obesity-related diseases.^5^

A second review was conducted on epigenetic markers, including DNA methylation and micro-RNA.^7^ High heterogeneity of the findings was noted and no strong inferences can be drawn. The temporal sequence between epigenetic changes and onset of childhood obesity is uncertain; however, as observed in adults, the available evidence suggests that DNA methylation changes are a consequence of adiposity rather than a cause.^8,9^ If obesity causes epigenetic changes, then epigenetics may fall on the causal pathway between obesity and obesity-related outcomes, as already suggested in previous children^10^ and adult studies.^11^

Overall, a conclusion of the systematic reviews is that molecular or metabolic research currently does not make consistent contributions to policy in terms of interventions to prevent obesity.

## Findings from the STOP consortium

An exposome approach linking dietary habits with the outcomes of childhood overweight and obesity, via intermediate omic markers, has been used for the first time in the large STOP consortium.

## External exposome: diet in children

Rather than performing an exposome-wide agnostic investigation on single foods (an approach with limitations), we have reclassified food items according to their degree of processing. Over the past decades diets, including in children, have shifted toward the consumption of ultra-processed foods (UPFs). According to the Nova food processing classification system, UPF are defined as foods and drinks that are industrially produced, which include substances derived from foods but not used in culinary preparations, such as hydrogenated fats and cosmetic additives. Examples of UPF include carbonated soft drinks, many ready meals, mass-produced packaged breads, and most breakfast cereals and are typically characterized by higher energy density and lower nutritional quality than minimally processed foods. Recent studies indicate that over 60% of total calories consumed among children in the United Kingdom and in the United States are from UPFs.^12,13^ The Nova classification has been instrumental in allowing the categorization of foods beyond a previously limited focus on nutrients. Assessment of UPF intake requires good dietary data and more granularity than usually available but efforts are underway to produce new measurement tools. Epidemiological evidence on the negative health impacts of UPF consumption has grown rapidly, but this is mainly focused on adults.^14,15^

## UPF and adiposity trajectories

To examine the effects of UPF consumption on obesity risk in children, we assessed longitudinal associations between UPF consumption and adiposity trajectories from childhood to early adulthood, among 9025 children participating in the British Avon ALSPAC, followed up from 7 to 24 years of age. Among those in the highest quintile of UPF consumption (>58% UPF/total calories) compared with their lowest quintile counterpart (<30% UPF/total calories), BMI increased by an additional 0.06 (95% confidence interval [CI], 0.04–0.08) per year with similar results for other measures of adiposity. Models were robust to adjustment for multiple factors including the child’s total energy intake and socio-economic factors including the Index of Multiple Deprivation, marital status of parents, maternal education, and UK National Statistics Socioeconomic Classification. Notably, we found that *UPF intake was strongly socially patterned*, finding a trend for increased consumption of UPF across all indicators examined. The work highlights the role that consumption of UPF may play in the stark social disparities in obesity rates observed today.^16^

### Internal exposome

An overview of the results concerning internal exposome research in STOP is shown in Table 1.

## Cord blood metabolic signatures predictive of childhood overweight and rapid growth

Metabolomics may identify biological mechanisms that increase the risk of overweight and obesity among children. In the STOP consortium, we investigated the cord blood metabolomic profiles of rapid growth in infancy and overweight in early childhood in four European birth cohorts (INMA, Rhea, Piccolipiù, and ENVIRONAGE combined together in the EXPOsOMICS study).^18^ Untargeted liquid chromatography-mass spectrometry (LC-MS) was applied in cord blood from around 400 newborns. Rapid weight growth in the first year of life and overweight in childhood (mean age 5.4 years) were defined according to WHO growth charts. We analyzed associations for rapid growth and overweight among over 4500 metabolic features, correcting for false discovery rate at 5%. We identified three metabolites associated with rapid growth and eight metabolites associated with overweight. Higher levels of cholestenone, a cholesterol derivative produced by microbial catabolism, were predictive of rapid growth. Lower levels of the BCAAs valine and leucine were predictive of overweight in childhood. Multivariate prediction models including identified metabolites showed good prediction of included outcomes with area under the receiver operator curve values of 0.77 for rapid growth and 0.82 for overweight, compared with 0.69 and 0.69, respectively, for models using traditional risk factors alone.

## Epigenetics: methylation-wide association study

The aim of our epigenetic analyses in STOP was to investigate the associations between blood DNA methylation at birth and rapid growth in the four EXPOsOMICS cohorts as above, plus an additional subset of ENVIRONAGE, GENERATION XXI (GXXI), and ALSPAC cohorts.^20^

For approximately 2000 children, cord blood DNA methylation was measured using Infinium arrays. Rapid weight growth in the first year of life and overweight in childhood (between 4 and 8 years) were defined as before. Epigenome-wide association studies for rapid growth were performed using multiple-adjusted logistic mixed effect models and then meta-analyzed. We found 47 Cytosine-phosphate-guanine (CpG) sites to be associated with rapid growth including three CpGs annotated to genes involved in adipocytes differentiation (cg14459032, cg25953130 annotated to *ARID5B* gene, and cg00049440 annotated to *KLF9* gene). Sixteen differentially methylated regions (DMRs) were identified as associated with rapid growth, one of which on the *AURKC* gene (involved in regulation of the mitotic cell division process) was also associated with childhood obesity between 4 and 8 years.

In spite of some suggestive findings (particularly based on the consistency between DNA methylation and transciptomics), evidence on the role of epigenetics in childhood overweight or obesity is so far limited and further studies are needed.

## Systems biology: multiomic analysis and birthweight

Multiomic analysis, that is, based on multiple measurements of changes in different categories of molecules, has been published in the STOP consortium.^17^ To investigate the systems biology of birthweight, we cross-sectionally integrated the methylome, the transcriptome, the metabolome, and a set of inflammatory proteins measured in cord blood samples, collected from four EXPOsOMICS birth cohorts as above (ENVIRONAGE, Rhea, INMA, and Piccolipiù). The analysis revealed that the set of metabolome, proteome, and methylome signatures of birthweight has seven signals in common, including three metabolites (including plasmalogens), two CpGs (on the *DHCR24* and *SC4MOL* genes), and two proteins (*periostin* and *CCL22*). Overall, the omics integration indicated a central role of cholesterol metabolism; therefore, we explored the association of cholesterol levels in cord blood with birthweight in the ENVIRONAGE cohort (*n* = 1097), where cholesterol fractions were measured independently of metabolomics. We found that higher birthweight was associated with increased high-density lipoprotein cholesterol and that high-density lipoprotein cholesterol was lower in small versus large for gestational age newborns.

The study suggests that an integration of different omic layers can assist in generation of new hypotheses regarding biological pathways. *Cholesterol metabolism* measured in cord blood may play a mechanistic role in birthweight, though it is not clear whether this is due to environmental (dietary) or genetic influences.

### UPF and metabolic profiles

**Figure 1:**
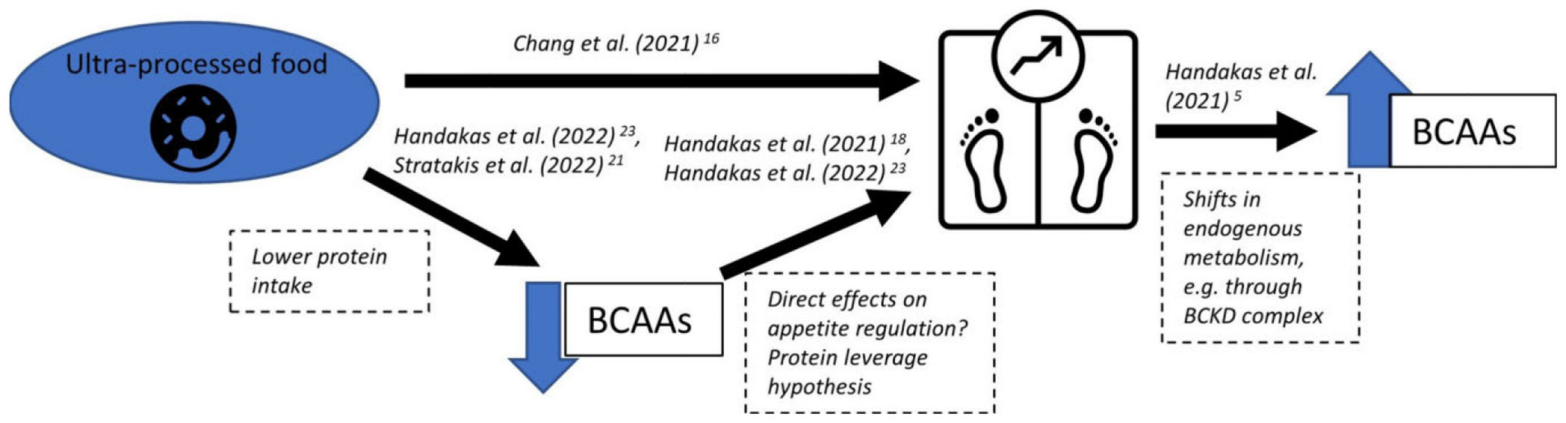
Schematic overview of the BCAA paradox. Black arrows represent associations reported in studies discussed in this article. Blue arrows represent decreased or increased BCAA levels. BCKD = branched-chain α-ketoacid dehydrogenase complex.

To elucidate the mechanisms underlying the association we found between UPF consumption and adiposity accumulation, we further analyzed the metabolic profiles of UPF consumption, using ^1^H nuclear magnetic resonance spectroscopy (NMR) within the ALSPAC cohort. This molecular signature of systemic metabolism consisted of 232 metabolic traits. We investigated the association between UPF consumption (as % of total energy intake) and themetabolome using multiple-adjusted linear regression models at 7 years of age. In the analysis of blood samples of over 4000 children, we found that a diet with a higher proportion of UPF was negatively associated with omega-3 fatty acids, phenylalanine, tyrosine, and BCAAs leucine, valine, and isoleucine. Box 1 and Figure 1 further develop the BCAA hypothesis and highlight a paradox in the findings. Monounsaturated fatty acids (MUFAs), citrate, glutamine, and creatinine were positively associated with UPF consumption. Additionally, negative associations were found for cholesterol and various lipoprotein subclasses.^23^

Children who consumed a greater proportion of UPF had lower reported intakes of proteins, fat, and micronutrients, and greater reported intake of carbohydrate and sugars. The association of UPF with lower reported intake of saturated fats and cholesterol was in contrast to South American studies,^24^ but was confirmed by metabolic profiling that showed lower circulating levels of these lipids in association with UPF consumption. Mediation analysis by nutrient intake indicated that the lower blood levels of BCAAs in association with UPF partly resulted from lower consumption of protein containing foods. Citrate is a very efficient food flavoring agent and preservative and as such is one of the most commonly used additives in the food industry.^25^ We speculate that citrate levels may serve as a general marker of UPF intake, particualry since mediation analysis did not indicate the role of specific nutrient intake in the association with UPF.

## Diet quality and insulin secretion in children in the HELIX consortium

C-peptide concentration is a marker of endogenous insulin secretion with lower levels associated with higher risk of diabetes.^21^ To examine the associations of Mediterranean diet adherence and UPF consumption with urinary metabolic profiles and serum C-peptide concentrations in children, we studied 1147 children (mean age 7.9 years), from the HELIX exposome cohort.^21^ Mediterranean diet adherence was assessed using a predefined score (KIDMED). UPF intake was assessed based on the Nova system. Urine metabolomic profiles were measured using NMR and C-peptide concentrations with the multiplex Luminex system. Associations of Mediterranean diet and UPF with metabolome profiles and C-peptide were analyzed by using linear regression modeling adjusted for child body mass index and sociodemographic variables.

We found that both a higher KIDMED score and lower UPF score was associated with lower C-peptide levels. Compared with children at the lowest quartile of UPF intake (<18% of total daily food intake), those at the highest quartile (≥29% of total daily food intake) had a 46% higher concentration of C-peptide (95% CI: 8.1–97.3%), with a signiticat trend observed across quartiles. The urinary metabolomic analysis identified a panel of six metabolites predictive of UPF consumption. Although four of these were also predictive of lower KIDMED score, lower levels of valine and tyrosine, as also observed in the study in ALSPAC, were found to be specifically associated with UPF.

## Meet-in-the-middle

Establishing causal relationships between external and behavioral exposures and outcomes such as rapid growth, overweight, or obesity in children can be strengthened by exposome research. By finding intermediate markers that are associated with both exposures and outcomes, a “meet-in-the-middle” approach lends biological credibility to statistical associations.^28^ In addition, the meet-in-the-middle approach can link social circumstances with behaviors, internal changes, and disease onset.^29^

In one study, we used multiple mediation analysis to explore the ability of the identified cord blood metabolites in the metabolome-wide association study (MWAS) and rapid growth as multiple mediators in the prenatal propensity to childhood overweight, as a response to seven potential obesogenic prenatal factors (maternal education, pre-pregnancy maternal body mass index (BMI), maternal weight gain during the pregnancy, tobacco smoke during pregnancy, maternal age at delivery, gestational age, and parity).^19^ Our results provide evidence that seven metabolites, including *cholestenone, decenoylcarnitine* (*C10:1*), *phosphatidylcholine* (*C34:3*), *progesterone*, and three other unidentified metabolites, mediated the effect of maternal education, pregnancy weight gain, parity, and gestational age on rapid growth but not directly on childhood overweight. Rapid growth, in turn, mediated the effect of gestational age on childhood overweight. Applying a multiple mediation approach, we elucidated that rapid growth was the main contributor in the mediation of the effect of gestational age on childhood overweight and that the mediating role of metabolites was marginal.

### BCAA and adiposity trajectories

To understand the role of metabolic profiles in adiposity trajectories, we investigated longitudinal associations between baseline quartiles of metabolic features at 7 years and fat mass measured at until 17 years of age, controlling for baseline adiposity. Evidence for a dose response in fat mass accumulation per year across quartiles was observed for BCAAs isoleucine and leucine, phenylalanine, tyrosine, citrate, and MUFA as ratio to total fatty acids. Taken together with work on UPF and adiposity trajectories, it supports a role for these metabolites in the association between UPF consumption and fat mass accumulation.

The study demonstrates the metabolic effects of nutrientpoor diets and provides possible pathways underlying the harmful effects of UPF. However, these results need replication and have limitations in relation to the Nova classification of UPF and the underlying limited granularity of dietary information.

### BCAA and air pollution

Research on children adiposity has considered multiple exposures in addition to diet, using a meet-in-the middle approach.^22^ In a multi-center cohort of 1301 mother–child pairs, individual exposomes consisting of >100 chemical, physical, and lifestyle exposures assessed in pregnancy and childhood, have been associated with multi-omics profiles (methylome, transcriptome, metabolome, and proteins) in childhood. In total, 1170 associations have been identified, 249 in pregnancy and 921 in childhood, which revealed potential biological responses and sources of exposure. The methylome best captured the persistent influence of pregnancy exposures, including maternal smoking; while childhood exposures were associated with features from all omics layers, revealing novel signatures for indoor air quality, essential trace elements, endocrine disruptors, and weather conditions. In particular, several methylation or omic associations for *indoor air quality during childhood* were found, in contrast to the few associations found for outdoor air pollution. Indoor levels of PM_2.5_ absorbance, a marker of black/elemental carbon originating from combustion, were associated with decreased levels of serum branched amino acids (BCAA: isoleucine, leucine, and valine), acylcarnitine C4 (butyrylcarnitine), and two sphingolipids. Lower BCAA and acylcarnitines were associated with exposure to nearroadway air pollution among young participants with obesity.^30^ Associations between dysregulated metabolism of BCAAs and acylcarnitines with obesity and insulin resistance have been widely observed in animal and adult human studies.^31^

We propose that altered BCAA and acylcarnitine metabolism may be an important biomarker to study further in relation to indoor air pollution and subsequent development of cardio-metabolic disease in later life. An association between indoor air pollution and increased child BMI was previously reported in the HELIX study, independently of correlated exposures such as second-hand smoke and lower social class status,^32^ and also in the systematic review by Malacarne et al. summarized above.

The observations above require further consolidation and replication in multiple cohorts, though they can be used to suggest some goals for primary prevention.

## Assessment and policy suggestions

In our research, we have primarily focused on maternal factors in the prenatal analyses, mainly due to data availability, but the role of paternal factors is increasingly recognized in developmental research. Notwithstanding these limitations, we propose some highlights (Box 2) and suggestions for policy.

On the basis of the highlights in Box 2 and other evidence in the STOP consortium, our research suggests a few interventions that are supported by evidence and should be implemented to reduce childhood obesity:

1. Limit intake of UPF in infancy and childhood, by limiting the proportion of calories they represent in diet (ideally below 30%).
2. Diversify children diets, with emphasis on fresh food.
3. Create opportunities for physical activity, including urban planning (safe areas reserved to children, green spaces, blue spaces, biking lanes) and promotion of sport activities at school.

Policy actions to prevent childhood obesity go far beyond the specific topic of this article: for a summary of STOP results and suggestions for policy, we direct readers to the STOP Factsheets available at https://www.stopchildobesity.eu/wp-content/uploads/2022/12/ which in particular outline actions to improve diets and opportunities for physical activity among children.

### Perspectives

We have proposed an exposome-based approach to strengthen causal claims in observational studies. Our series of studies in the STOP consortium, in addition to previous evidence, seems to suggest that by linking external exposures with outcomes via intermediate agnostic omic investigations can be rewarding and lead to plausible causal associations. Future research in the field of UPF and childhood obesity should aim to replicate our findings and clarify some biological inconsistencies. However, we believe that the bulk of existing evidence on UPF, including studies in adults, is sufficient to regulate UPF intake in infancy.

## Figures and Tables

**Table 1:** Summary of relevant internal exposome papers discussed in this article

References	Design	Methods	Cohorts	Age range (years)	Relevant findings
Handakas et al.^5^	Systematic review	Qualitative synthesis of 41 studies of metabolomics and childhood obesity	–	0–18	A consistent metabolic profile of childhood obesity was observed including BCAAs, AAAs, carnitines, lipids, and steroids. Few prospective studies identified
Alfano et al.^7^	Systematic review	Qualitative synthesis of 121 studies of epigenetics and childhood obesity	–	0–18	High heterogeneity of the findings, with evidence more strongly supporting an influence of adiposity on DNA methylation rather than vice versa
Alfano et al.^17^	Cross-sectional multi-cohort	Multi-omic analysis of birthweight	ENVIRONAGE (Be), Rhea (EL), INMA (SP), Piccolipiù (IT)	0	Omics integration indicated a central role of cholesterol metabolism in variance in birthweight
Handakas et al.^18^	Prospective multi-cohort	Untargeted LC-MS metabolomic analysis in cord blood with rapid growth/childhood obesity	ENVIRONAGE (Be), Rhea (EL), INMA (SP), Piccolipiù (IT)	0–6	Higher levels of cord blood cholestenone and lower levels of BCAAs were predictive of rapid growth and overweight in childhood, respectively
Alfano et al.^19^	Prospective multi-cohort	Analysis of cord blood metabolic markers of rapid growth/ childhood obesity as mediators of prenatal risk factors	ENVIRONAGE (Be), Rhea (EL), INMA (SP), Piccolipiù (IT)	0–6	Seven metabolites were identified as mediators of prenatal risk factors and rapid growth
Alfano et al.^20^	Prospective multi-cohort	EWAS analysis of cord blood methylation and rapid growth/ childhood obesity	ENVIRONAGE (Be), Rhea (EL), INMA (SP), Piccolipiù (IT), Gen21 (PT), ALSPAC (United Kingdom)	0–6	47 CpGs and 16 DMRs in cord blood were associated with rapid growth. One DMR in AURKC gene also was associated with childhood obesity
Stratakis et al.^21^	Cross-sectional multi-cohort	Urinary NMR metabolomic and insulin resistance analysis of UPF and Mediterranean diet	Rhea (EL), INMA (SP), BiB (United Kingdom), Moba (NO), KANC (LT)	5–12	Higher UPF consumption associated with higher C-peptide (a marker of insulin resistance) and a metabolic prolife including lower levels of BCAAs
Maitre et al.^22^	Cross-sectional and prospective multi-cohort	Childhood multi-omic profiles of prenatal and child external exposome	Rhea (EL), INMA (SP), BiB (United Kingdom), Moba (NO), KANC (LT)	5–12	Indoor air pollution (previously associated with childhood obesity) associated with lower levels of BCAAs
Chang et al.^16^	Prospective	Analysis of UPF consumption and adiposity trajectories during childhood	ALSPAC (United Kingdom)	7–24	Higher UPF consumption at 7 years associated with greater subsequent fat mass accumulation, independently of total energy consumption and socio-demographic factors
Handakas et al.^23^	Prospective	Blood NMR metabolomic analysis of UPF consumption and role in adiposity trajectories during childhood	ALSPAC (United Kingdom)	7–17	Higher UPF consumption associated with multiple metabolic markers, also associated with greater subsequent fat mass accumulation, including lower levels of BCAAs

## Data Availability

No data were used in writing this review.

## Box 1 The evidence on BCAA involvement in adiposity

BCAAs (leucine, isoleucine, and valine) are essential amino acids found in protein-containing foods that have both direct and indirect nutrient signaling effects. We observed in two STOP studies^18,23^ that lower blood BCAAs are predictive of later obesity risk, and furthermore a diet rich in UPF was also associated with lower BCAAs levels in separate STOP studies.^21,23^ Mulitple intervention studies and animal studies have suggested that greater intake of BCAAs has beneficial effects on appetite and metabolic parameters.^26,27^ These positive effects may potentially be mediated through direct signaling actions on hypothalamic and brainstem processes involved in satiety.^26^ Lower BCAAs intake could therefore increase risk for overweight through intermediate processes such as control of food intake. However, our observations are seemingly at odds with those of previous investigations as summarized in our systematic review on metabolomics that consistently showed higher BCAA blood levels among children already with obesity.^5^ The observed increases associated with obesity likely result from physiological shifts such as through branched-chain α-ketoacid dehydrogenase complex (BCKD) complex activity, which lowers BCAA catabolism and clearance,^27^ whereas mediation analysis indicated that the lower blood levels observed in association with UPF appear to be driven by lower intake of protein-containing foods, rather than endogenous mechanisms. This BCAA paradox is graphically expressed in Figure 1.

## Box 2 Highlights of the findings and research needs

Our literature reviews on metabolomics and epigenetics suggest that the evidence is still immature and too heterogeneous to draw strong inferences, though metabolomics in cord blood points to a relationship between high cholestenone and infant rapid growth, and between *lower BCAAs intake* and obesity risk in childhood.

Our biomarker-based studies in the STOP consortium strongly suggest (in several ways, including mediation analysis) BCAAs as mediators of risk factors for childhood overweight/obesity. This is at odds with the systematic review above, mainly based on cross-sectional studies of child already with overweight/obesity. However, the STOP original observations are consistent with intervention studies and animal studies showing that higher intake of BCAAs has positive impact on parameters including body composition, glycemia, and satiety. BCAAs intake could influence later propensity for overweight through causal processes such as control of food intake, contributing to effects of dietary patterns on weight gain. However, mechanisms still need clarification.

Our epigenetics-based study in cord blood shows that DNA methylation of regions of DNA and to a lesser extent single CpGs located on genes involved in adipocytes differentiation were associated with rapid weight growth in infancy; one of the identified regions, on the *AURKC* gene (involved in regulation of the mitotic cell division process), was associated with obesity in childhood. However, evidence on the role of epigenetics in childhood overweight or obesity is so far limited and further studies are needed.

Concerning food, of particular concern is the trend of increasing intake of UPF, including among children. Research from the ALSPAC cohort has shown a relationship between UPF intake and trajectories in childhood adiposity. In the ALSPAC^23^ and HELIX^21^ studies, UPF was related to lower blood or urinary levels of BCAAs, providing a potential mechanism underlying control of food intake and obesity risk. While it is unclear if these associations represent a causal relationship, it is likely that BCAAs levels are indicative of overall diet quality such as protein intake. The protein leverage hypothesis proposes that a lower levels of protein may reduce feelings of satiety leading to overeating.^33^ Several mechanisms have been proposed to explain the impact of UPF on obesity and overweight in adults, including changes in nutrient intake, changes in appetite (possibly mediated by gut hormones), or the role of additives. In particular, experimental studies on food consumption indicate that UPFs have low satiety potential and induce high glycaemic responses.^15,34^

While further research is needed on the role of UPF, especially on mechanistic pathways, evidence on their harmful effects is sufficient to limit their intake in children.

Socio-economic position is a powerful driver of overweight and obesity in children, probably through several pathways and mechanisms, including lower opportunities for physical activity (also related to the built environment) and poorer dietary habits (including lower intake of BCAA and higher intake of UPF).

## References

[1] WHO (2017).

[2] Baidal JAW, Locks LM, Cheng ER, Blake-Lamb TL, Perkins ME, Taveras EM (2016). Risk factors for childhood obesity in the first 1,000 days: a systematic review. Am J Prev Med.

[3] Malacarne D, Handakas E, Robinson O (2022). The built environment as determinant of childhood obesity: A systematic literature review. Obes Rev.

[4] Fernández-Barrés S, Robinson O, Fossati S (2022). Urban environment and health behaviours in children from six European countries. Environ Int.

[5] Handakas E, Lau CH, Alfano R (2022). A systematic review of metabolomic studies of childhood obesity: state of the evidence for metabolic determinants and consequences. Obes Rev.

[6] Rangel-Huerta OD, Pastor-Villaescusa B, Gil A (2019). Are we close to defining a metabolomic signature of human obesity? A systematic review of metabolomics studies. Metabolomics.

[7] Alfano R, Robinson O, Handakas E, Nawrot TS, Vineis P, Plusquin M (2022). Perspectives and challenges of epigenetic determinants of childhood obesity: A systematic review article. Obes Rev.

[8] Sun D, Zhang T, Su S (2019). Body mass index drives changes in DNA methylation: A longitudinal study. Circ Res.

[9] Wahl S, Drong A, Lehne B (2017). Epigenome-wide association study of body mass index, and the adverse outcomes of adiposity. Nature.

[10] Reed ZE, Suderman MJ, Relton CL, Davis OS, Hemani G (2020). The association of DNA methylation with body mass index: distinguishing between predictors and biomarkers. Clin Epigenet.

[11] Campanella G, Gunter MJ, Polidoro S (2018). Epigenome-wide association study of adiposity and future risk of obesity-related diseases. Int J Obes (Lond).

[12] Onita BM, Azeredo CM, Jaime PC, Levy RB, Rauber F (2021). Eating context and its association with ultra-processed food consumption by British children. Appetite.

[13] Neri D, Martinez-Steele E, Monteiro CA, Levy RB (2019). Consumption of ultra-processed foods and its association with added sugar content in the diets of US children, NHANES 2009–2014. Pediatr Obes.

[14] Monteiro CA, Cannon G, Moubarac J-C, Levy RB, Louzada MLC, Jaime PC (2018). The UN decade of nutrition, the NOVA food classification and the trouble with ultra-processing. Public Health Nutr.

[15] Monteiro CA, Cannon G, Levy RB (2019). Ultra-processed foods: What they are and how to identify them. Public Health Nutr.

[16] Chang K, Khandpur N, Neri D (2021). Association between childhood consumption of ultraprocessed food and adiposity trajectories in the avon longitudinal study of parents and children birth cohort. JAMA Pediatr.

[17] Alfano R, Chadeau-Hyam M, Ghantous A (2020). A multi-omic analysis of birthweight in newborn cord blood reveals new underlying mechanisms related to cholesterol metabolism. Metabolism.

[18] Handakas E, Keski-Rahkonen P, Chatzi L (2021). Cord blood metabolic signatures predictive of childhood overweight and rapid growth. Int J Obes.

[19] Alfano R, Plusquin M, Robinson O (2022). Cord blood metabolites and rapid postnatal growth as multiple mediators in the prenatal propensity to childhood overweight. Int J Obes (Lond).

[20] Alfano R, Zugna D, Barros H (2023). Cord blood epigenome-wide meta-analysis in six European-based child cohorts identifies signatures linked to rapid weight growth. BMC Med.

[21] Stratakis N, Siskos AP, Papadopoulou E (2022). Urinary metabolic biomarkers of diet quality in European children are associated with metabolic health. Elife.

[22] Maitre L, Bustamante M, Hernández-Ferrer C (2022). Multi-omics signatures of the human early life exposome. Nat Commun.

[23] Handakas E, Chang K, Khandpur N (2022). Metabolic profiles of ultraprocessed food consumption and their role in obesity risk in British children. Clin Nutr.

[24] Araya C, Corvalán C, Cediel G, Taillie L, Reyes M (2021). Ultra-processed food consumption among chilean preschoolers is associated with diets promoting non-communicable diseases. Front Nutr.

[25] Evans G, de Challemaison B, Cox DN (2010). Consumers’ ratings of the natural and unnatural qualities of foods. Appetite.

[26] Lynch CJ, Adams SH (2014). Branched-chain amino acids in metabolic signalling and insulin resistance. Nat Rev Endocrinol.

[27] Pallares-Méndez R, Aguilar-Salinas CA, Cruz-Bautista I, del Bosque-Plata L (2016). Metabolomics in diabetes, a review. Ann Med.

[28] Chadeau-Hyam M, Athersuch TJ, Keun HC (2011). Meeting-in-the-middle using metabolic profiling—a strategy for the identification of intermediate biomarkers in cohort studies. Biomarkers.

[29] Vineis P, Barouki R (2022). The exposome as the science of social-to-biological transitions. Environ Int.

[30] Chen Z, Newgard CB, Kim JS (2019). Near-roadway air pollution exposure and altered fatty acid oxidation among adolescents and young adults—the interplay with obesity. Environ Int.

[31] Newgard CB (2017). Metabolomics and metabolic diseases: Where do we stand?. Cell Metab.

[32] Vrijheid M, Fossati S, Maitre L (2020). Early-life environmental exposures and childhood obesity: An exposome-wide approach. Environ Health Perspect.

[33] Steele EM, Raubenheimer D, Simpson SJ, Baraldi LG, Monteiro CA (2018). Ultra-processed foods, protein leverage and energy intake in the USA. Public Health Nutr.

[34] Fardet A (2016). Minimally processed foods are more satiating and less hyperglycemic than ultra-processed foods: A preliminary study with 98 ready-to-eat foods. Food Funct.

